# Polygenic risk moderation of stressful life events in alcohol use disorder severity

**DOI:** 10.1186/s12888-026-07920-6

**Published:** 2026-02-23

**Authors:** Zena Agabani, Nzaar Al Chalabi, Vincenzo De Luca, Bernard Le Foll, Ahmed N. Hassan

**Affiliations:** 1 Campbell Family Mental Health Research Institute, Centre for Addiction and Mental Health, Toronto, ON M6J 1H4 Canada; 2 Department of Pharmacology and Toxicology, Faculty of Medicine, University of Toronto, Toronto, ON M5S 1A1 Canada; 3 Department of Psychiatry, Faculty of Medicine, University of Toronto, Toronto, ON Canada; 4 Institute of Medical Sciences, University of Toronto, Toronto, ON M5S 1A1 Canada; 5 Waypoint Research Institute, Waypoint Centre for Mental Health Care, 500 Church Street, Penetanguishene, ON L9M 1G3 Canada; 6 Department of Family and Community Medicine, Temerty School of Medicine, University of Toronto, Toronto, ON Canada

**Keywords:** Alcohol use disorder, Social stress, Polygenic risk, NESARC, Alcohol, GxE, Propensity score

## Abstract

**Background:**

Advances in gene–environment (GE) research underscore the need for novel statistical methods to clarify how social stress and genetic liability jointly influence Alcohol Use Disorder (AUD). This study examined the effect of Stressful Life Events (SLEs) on past-year DSM-5 AUD severity using propensity score matching and tested whether Polygenic Risk Scores (PRS) moderated the association between stress exposure and AUD severity.

**Methods:**

A secondary analysis was performed using the data from National Epidemiologic Survey on Alcohol and Related Conditions III (NESARC III; *N* = 36,309) and the newly available NESARC III genomic dataset (*N* = 22,848). Of 10,775 self-reported White subjects, individuals in the high-stress group (i.e., more than two stressors) (*N* = 4436) were matched to those in the low-stress group (*N* = 5048) using propensity score matching. Further, a moderation analysis was conducted to evaluate the interaction between PRS and SLE.

**Results:**

Exposure to two or more SLEs in the past year significantly increased the odds of AUD severity after covariate adjustment (estimate: 0.37; 95%CI: 0.27–0.48; t-value: 6.96; *p* < 0.001). PRS was independently associated with higher AUD criteria counts after adjustment (estimate: 0.23; 95%CI: 0.07–0.39; t-value: 2.87; *p* = 0.01). PRS significantly moderated the relationship between the stress exposure and the AUD severity (interaction effect = 0.30; 95% CI: 0.06–0.54; t = 2.47; *p* = 0.01), indicating stronger stress-related effects among individuals with higher genetic risk. Additional sensitivity analyses suggested that PRS moderation by stress exposure is small and sensitive to model specification.

**Conclusions:**

This novel GE interaction model provides evidence that polygenic risk can potentiate the effects of high-stress exposure on the severity of AUD, thereby advancing post-GWAS modeling efforts and support precise identification of high-risk subpopulations for targeted prevention and intervention.

**Clinical trial number:**

Not applicable.

## Introduction

Alcohol use disorder (AUD) is a major public health issue with substantial health, social, and economic consequences [1]. In the United States, excessive alcohol use contributes to over 178,000 deaths annually and remains a leading preventable cause of mortality [2]. Despite demographic declines in alcohol consumption, an estimated 27.1 million adults aged 18 years and older (10.3%) meet criteria for AUD in the past year [3].

While a large proportion of the population is exposed to alcohol and other addictive substances, only a subset of individuals transition from recreational use to addiction [4]. This divergence highlights critical gaps in understanding the mechanisms that drive progression to problematic drinking. Emerging trends in alcohol consumption warrant personalized diagnostic frameworks that account for individual differences in vulnerability, including genetic and environmental factors that shape risk trajectories. Nationally representative research is needed to elucidate these mechanisms, clarify behavioral trends, and inform targeted prevention strategies for AUD.

The high incidence of comorbidities between AUD and stress-related disorders suggests a shared pattern of psychopathology that is not disorder-specific [5]. Therefore, a more practical approach to understanding the etiology underlying AUD is to evaluate shared risk factors, such as stress exposure. Stress exposure is a well-established social determinant of health in the context of addiction and psychiatry. One common way to operationalize stress exposure is through stressful life events (SLEs), which refer to acute, discrete events that challenge an individual’s coping abilities and increase the risk for psychopathology and maladaptive behaviors [6]. SLEs can encompass a wide range of experiences, including job loss, interpersonal conflicts, serious illness, or the death of a loved one, and definitions vary depending on the measurement approach. In the NESARC dataset, SLEs are assessed as specific adverse experiences occurring within the past 12 months, providing a standardized yet broad measure of acute stress exposure. Approximately 60–70% of people report experiencing at least one SLE annually [6]. Using data from Wave 3 (2012–2013) of the NESARC survey (NESARC-III), Verplaetse et al. (2018) found that males exposed to two or more SLEs had increased odds of developing a new AUD, while females exposed to two or more SLEs showed the greatest odds of a new AUD [7]. Notably, SLE exposure also impacted substance use transitions, as experiencing two or more SLEs was associated with greater odds of ongoing AUD [7].

Gene–environment (GE) interactions have been used to explain diverse human phenotypes [7]. The stress diathesis model of psychopathology, also known as the vulnerability stress model, states that the onset of psychological disorders is a result of an environmental stressor that interacts with an individual’s inherent vulnerability to develop a disorder (diathesis) [8]. Numerous GE studies have shown how adverse events have the potential to exacerbate genetic vulnerability to AUD [9, 10]. Recent advances in genome-wide association studies (GWAS) of alcohol use disorder (AUD) have increasingly employed dimensional measures of alcohol consumption, revealing distinct genetic architectures underlying normative versus problematic use [11]. Genome-wide–by–environment interaction (GWEIS) studies further suggest that environmental exposures shape genetic risk; however, these findings are often constrained by limited sample sizes and restricted demographic representation [11].

As a result, post-GWAS approaches and genetically informative models are needed to better characterize heterogeneous and at-risk subpopulations. Polygenic methods have proven particularly useful in studying complex disorders, offering predictive power beyond that of individual genetic variants [12]. In this context, polygenic risk scores (PRS) have emerged as a promising tool for modeling the genetic architecture of substance use disorders, enabling the identification of individuals at elevated risk by aggregating many variants of small effect [13, 14]. PRSs are derived from GWAS summary statistics and have been consistently associated with AUD-related phenotypes, particularly among individuals of European ancestry [13, 15–18]. Despite these advances, existing AUD–PRS research has largely examined genetic risk in isolation, with limited integration of clinical and demographic variables or rigorous causal-inference methods. In particular, few studies have incorporated propensity score–based approaches to evaluate shared genetic and environmental risk, limiting the translational relevance of PRS findings.

In the present study, we introduce a novel analytical framework that integrates genetic and environmental risk factors to examine AUD severity. First, we estimated the effect of stressful life event (SLE) exposure on AUD severity using propensity score methods that accounted for established confounders. Second, we constructed a PRS for AUD severity based on item-level GWAS data and evaluated its independent association with AUD severity. Finally, we tested whether polygenic risk moderates the relationship between SLE exposure and AUD severity. Together, these analyses aim to clarify how polygenic liability and stress exposure interact to shape AUD outcomes.

## Methods

### Data sources

We conducted a secondary analysis using data from the third wave of the National Epidemiological Survey for Alcohol and Related Conditions (NESARC III; *N* = 36,309), a cross-sectional epidemiological study conducted between 2012 and 2013 by the National Institute on Alcohol Abuse and Alcoholism (NIAAA) [20]. The population included non-institutionalized American adult civilians aged ≥ 18 years residing in the United States [20]. All participants completed an informed consent, and the research protocol received full ethical review approval from the National Institute on Alcohol Abuse and Alcoholism and Westat [20]. Multistage probability sampling was used to randomly select participants, with counties or groups of contiguous counties as primary sampling units, census-defined blocks or groups of blocks as secondary sampling units, and households within secondary units as tertiary sampling units. Primary and secondary sampling have been previously described [20]. The screener-level, person-level, and overall response rates were 72%, 84%, and 60%, respectively [20]. The data were adjusted for oversampling and weighted by post-stratification to represent the population and compensate for person-level non-responses.

#### AUD and psychiatric disorder diagnoses

Background information, alcohol consumption, criteria of AUD, and other clinical diagnoses, including mood disorders (i.e., major depression, bipolar disorder, or dysthymia/persistent depressive disorder), anxiety disorders (i.e., generalized anxiety disorder, panic disorder, agoraphobia, social anxiety, or specific phobia), personality disorders (i.e., schizotypal, borderline personality disorder, or antisocial personality disorder), and other substance use disorders (i.e., cannabis use, stimulant use, opioid use or sedative use disorders), data were collected from a structured interview (Alcohol Use Disorder and Associated Disabilities Interview Schedule-5) [20]. This test has fair-to-excellent test–retest reliability in the general population, according to the Diagnostic and Statistical Manual of Mental Disorders, Fifth Edition (DSM-5) [20]. Adverse childhood traumatic experiences, including sexual abuse, physical abuse, emotional abuse, emotional neglect, and physical neglect, were measured using 13 Likert-scale questions from the Childhood Trauma Questionnaire (21–23], and childhood abuse factors were measured using the Conflict Tactics Scale [22]. These questions measure the frequency and intensity of nontraumatic experiences. Parents’ adverse experiences consisted of binary variables indicating the presence or absence of four adversities: going to jail, hospitalization due to mental illness, attempting suicide, or committing suicide. We chose the AUD severity (sum of the past-year AUD criteria), which is a continuous indicator, as our primary outcome.

#### SLEs

SLEs comprised 16 questions about stressful events that occurred in the past year, including stress from moving, job loss, job responsibilities, relationship loss, interpersonal conflict, financial difficulties, trouble with the law, home destruction, homelessness, and crime. The total number of stressful life events was calculated based on answers to the presence or absence of these questions. High stress was defined as two or more stressful events during the past year. Previous researchers have used this definition based on the distribution of SLEs that were positively skewed and to conduct a median split [7, 23]. SLE was divided into two groups only to apply propensity score matching that matches the two groups on known risk factors (please see details below).

#### GWAS

Our work builds on the GWAS from the recent investigations of Mallard et al. (2022), which identified candidate genes significantly associated with dimensional aspects of AUD, measured by the Alcohol Use Disorders Identification Test (AUDIT). Mallard et al. (2022) represents the largest item-level GWAS to date related to AUDIT (*N* = 160,824) [24]. AUDIT is a 10-item scale used to screen to alcohol-related problems and has shown reasonable performance in screening for DSM-5 AUD [25]. However, using total scores without item-level modeling can introduce errors due to the lack of item-specific weights [25]. The AUDIT-Consumption (AUDIT-C) subscale sums items 1–3, focusing on the quantity and frequency of alcohol consumption, while the AUDIT-Problems (AUDIT-P) subscale sums items 4–6, capturing AUD symptoms [25]. As demonstrated by Mallard et al. (2022), the latent AUDIT-C factor captures shared variance most relevant for downstream genetic prediction. The latent AUDIT-C factor was chosen in this study to maximize genetic signal strength, which shows higher heritability and greater GWAS power compared to problem-related measures [26]. Consistent with prior work, this factor shows only moderate genetic correlation with AUD diagnosis; however, its substantially larger discovery sample size and higher SNP heritability make it more suitable for PRS derivation in the present study.

The GWAS used discovery samples from three population-based cohorts: the UK Biobank (*N* = 147,267), the Netherlands’ Twin Register (*N* = 9,975), and the Avon Longitudinal Study of Parents and Children (*N* = 3582). The Miami plot of the discovery GWAS for AUDIT-C displays SNPs contributing to study-specific signals (suggestive significance, *p* = 1 × 10^− 5^). SNP-level summary statistics were provided upon request and used to determine secondary PRS.

Standard Quality Control (QC) measures were conducted on the Mallard (2022) summary statistics (base data) to yield the most powerful GWAS results and ensure PRS validity and reliability [26]. In addition to the QC measures conducted in the GWAS (refer to Mallard et al., 2020), we removed SNPs with low MAF (< 0.01), and low imputation quality (INFO) (threshold = 0.8) [26]. We removed strand-ambiguous SNPs (i.e. those with complementary alleles, either C/G or A/T SNPs) and duplicate SNPs (indicative of multiallelic markers) in both the base and target data. We also resolved mismatched SNPs names between the two datasets. The unresolved, mismatching SNPs were removed as well. We removed SNPs with low genotyping rate (0.01), low minor allele frequency (0.01) out of Hardy-Weinberg Equilibrium (1 × 10^− 6^), removing SNPs with low genotyping rate (0.1). At total of 6,377,329 SNPs passed quality control measures to be accounted for in PRS analysis.

#### Target dataset

This study utilized the newly available NESARC-III genomic dataset. Of the 36,309 NESARC-III respondents, saliva samples were obtained from 23,360 subjects [21]. This genetic sample is available to researchers at dbGaP (accession number: phs001590.v2.p1) [28]. Genotyping, data processing, and quality control (QC) of the samples have been reported previously [19].

DNA was extracted from saliva samples and genotyped using the Affymetrix Axiom^®^ Exome Array [19]. The array encompassed 319,283 SNPs, and an additional 103,404 custom SNPs were selected based on genes linked to addiction in a previous study [19]. QC to remove SNPs was performed using the standard Affymetrix SNPs polisher algorithm [19], yielding 396,581 SNPs for subsequent analyses. SNPs were excluded for deviation from Hady–Weinberg equilibrium (*p* < 1 × 10^− 7^) if their genotyping call rate was < 97% and if they were monomorphic or represented by a solitary heterozygote [19]. Further QC steps were performed on the samples, and samples from genetically related individuals (*N* = 824) and those from individuals with a genotyping call rate of < 97% (*N* = 188) were excluded. Overall, 22,848 samples representing 260,779 SNPs were included in the analysis [26]. Notably, 21,840 SNPs had a minor allele frequency (MAF) score of 0.01–0.05 [19].

#### PRS derivation

Our series of computations were performed using the Centre of Addiction and Mental Health Specialized Computing Cluster (SCC).

#### Imputation

Genotype imputation was performed on our NESARC-III target dataset using the Michigan Imputation Server [29]. EAGLE2 was used to phase each chromosome uploaded to the server [30]. Minimac4 was used to impute genotypes, using the 1000 Genomes Project Phase 3 data (build 37) as the reference panel [29]. Post-imputation quality control measures were conducted on the target dataset, to the standard applied in our GWAS study [27]. We implemented an Imputation Accuracy threshold (R^2^) of 0.3 and removed SNPs with low Minor Allele Frequency (MAF) (< 0.01).

#### Population stratification

Only European ancestry (EA) samples were included in the PRS analysis because of the limited non-European ancestry samples used in our reference GWAS and the complex linkage disequilibrium structures present in admixed populations (Latinx, African American, and Aboriginal) [14, 33]. A Principal Component Analysis (PCA) was conducted to estimate global ancestry and plot genetic relatedness to understand the population’s genetic structure [31, 32]. We selected individuals in the NESARC-III clinical dataset who self-reported their race as white (non-Hispanic) and provided a genetic sample (*N* = 11,815). In addition, we selected individuals who reported drinking at least one alcoholic drink in their lives (*N* = 10,775). Outlier identification steps were conducted to confirm European ancestry. Outlier samples were defined as individuals whose ancestry was at least “6 standard deviations” (SD) away from the mean PC1-10 [32].

#### PRS calculation

Standard Quality Control (QC) measures were conducted on AUDT-C summary statistics (base data) to yield the most powerful GWAS results and ensure PRS validity and reliability [26]. In addition to the QC measures conducted in the GWAS (refer to Mallard et al., 2020), we removed SNPs with low MAF (< 0.01), and low genotyping rate (INFO) (threshold = 0.8) [27]. We removed strand-ambiguous SNPs (i.e. those with complementary alleles, either C/G or A/T SNPs) and duplicate SNPs (indicative of multiallelic markers) in both the base and target data. We also resolved mismatched SNPs names between the two datasets. The unresolved, mismatching SNPs were removed as well. We removed SNPs with low genotyping rate (0.01), low minor allele frequency (0.01) out of Hardy-Weinberg Equilibrium (1 × 10-6), removing SNPs with low genotyping rate (INFO) threshold = 0.8. At total of 6,377,329 SNPs passed quality control measures to be accounted for in PRS analysis.

The PRSice-2.3.3 software package was used to calculate PRS [33]. PRSice implements P-value thresholding, known as the standard “C + T method” [34]. PRSice-2 performs clumping to remove regions of high LD by default [34]. Sex and the first 10 principal components were included as covariates.

### Propensity score matching variables

This study used propensity score matching to ensure that the high- and low-stress groups were as similar as possible [35]. Matching was based on the variables that created a propensity score for the probability of experiencing adult stress or AUD. These matching variables are known risk factors for adult stress or AUD, including childhood maltreatment, which also help avoid measuring adult stress as a proxy for childhood stress.

The matching variables included sex, parents’ adverse experience (went to jail, hospitalization, died, divorced, attempted or committed suicide), personality disorders (antisocial, borderline, and schizotypal), childhood adverse events (total score of Likert scale of different adversities, including sexual abuse, physical abuse, physical neglect, or emotional abuse), prior to past-year mood disorders (major depression, bipolar disorder, or dysthymia/persistent depressive disorder), prior to past-year anxiety disorders (generalized anxiety disorder, social anxiety, panic disorder, agoraphobia, specific phobia), presence of any prior to past-year substance use disorders (i.e., alcohol use disorder, cannabis use disorder, stimulant use disorder, opioid use disorder, or sedative use disorders), age at onset of alcohol use, and birthplace (born in U.S. or non-U.S.) [23, 36–43]. Previous research shows that using many matching variables is crucial for achieving valid matches in propensity-score analyses [35]. Other known risk factors, such as marital status and education, which may have been affected by stress exposure, violate the propensity score assumption. Therefore, we controlled for these confounding variables in the final multivariable model, as suggested previously [35].

We implemented a “nearest” matching approach to estimate the propensity score, which includes setting all individuals exposed to high (exposure group) and low (control group) stress into a series of matched sets [35]. The nearest approach provides a good balance of covariates [35]. The analysis was performed “with replacement” using a 1:4 ratio [35] to ensure the best balance between the two groups. Of the 10,775 individuals, we matched individuals in the high-stress group (*N* = 4,436) to those in the low-stress group (*N* = 5,048). 1291 individuals from the low-stress group did not have a good match and were excluded. We assessed the average treatment effect, which estimates the average effect on the risk of AUD severity for individuals who have had many stressful events [35]. Further, we estimated the propensity score using logistic regression with stressful events as an outcome and the chosen covariates as predictors. Numerical and visual methods were used to confirm a good balance. For the numerical methods, we ensured that the standardized bias, the difference in means between the two groups divided by the SD, was < 0.25 [44], implying the standardized difference in means between the two groups to be > 0.25 SDs (Table 1). The propensity score was estimated using the MatchIt package in R [44, 45].

**Fig. 1:**
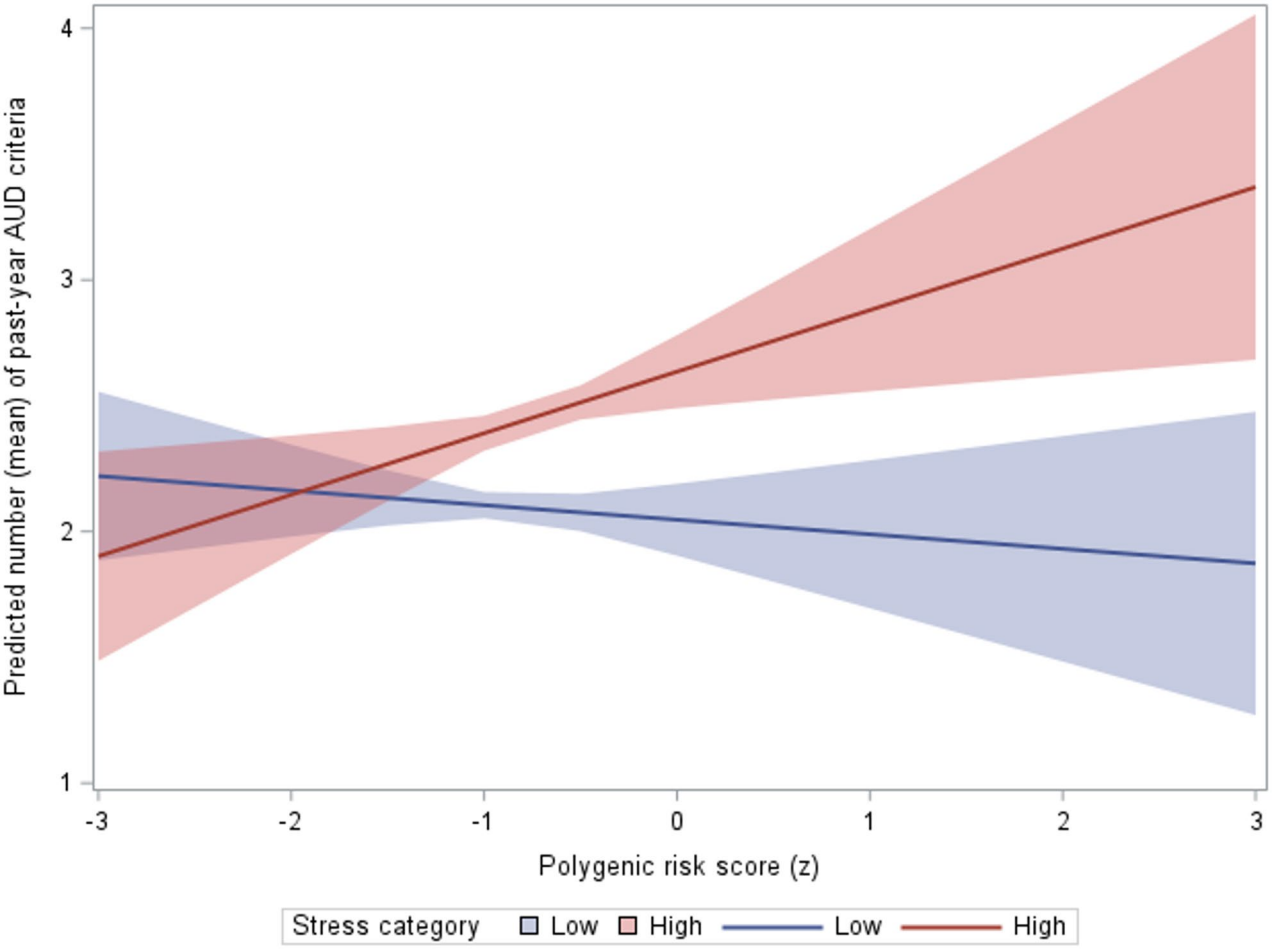
Interaction between the alcohol use disorder polygenic risk score (PRS) and stressful life events (SLE) in relation to past-year DSM-5 alcohol use disorder (AUD) criteria count. Lines show covariate-adjusted predicted mean AUD criteria across the standardized AUDIT-C–based PRS (x-axis) for low stress (0–1 SLE; solid blue) and high stress (≥ 2 SLE; solid red) in the matched analytic sample; shaded bands indicate 95% confidence intervals. Predicted AUD criteria increased more steeply with higher PRS in the high-stress group, consistent with a PRS × SLE interaction

**Table 1:** Clinical characteristics of the participants by stress category

Characteristic	Low stress (*N* = 5048) Mean (SE)	High Stress (*N* = 4436) Mean (SE)	*p*-value	Pearson Chi-square	Standardized mean differences before matching	Standardized mean differences after matching
Age	52.13 (0.38)	42.26 (0.30)	< 0.001		N/A	N/A
Age at first drink	18.53 (0.94)	18.60 (0.13)	0.6705		-0.2209	0.0088
Childhood adverse experiences (total score)	29.26 (0.28)	29.29 (0.22)	0.9379		0.2919	0.0020
Sex			0.7083	0.1616	0.0243	0.0083
Male	2354 (44.5)	1994 (45.0)				
Female	2694 (55.5)	2442 (55.0)				
Number of DSM-5 AUD criteria	2.04 (0.04)	2.66 (0.04)	< 0.001		N/A	N/A
	**N (% of total population**, ***N = 9484)***					
Born in the United States			0.0159	0.0159	0.0913	0.0224
Yes	4847 (51.9)	4295 (45.3)				
No	201 (1.3)	141 (1.5)				
Marital Status			< 0.0001	458.7340	N/A	N/A
Married	2649 (26.6)	1597 (16.8)				
Living with someone as if married	210 (2.4)	346 (3.6)				
Widowed	562 (5.2)	195 (2.1)				
Divorced	787 (9.6)	238 (2.5)				
Separated	94 (2.1)	238 (5.4)				
Never married	746 (8.3)	1196 (12.6)				
Any DSM-5 personality disorder			0.4249	1.2051	0.3582	0.0224
No	4578 (41.8)	3439 (36.3)				
Yes	470 (11.5)	997 (10.5)				
Parents’ adverse experiences			0.0521	0.8342	0.2474	0.0047
No	4469 (42.9)	3569 (37.6)				
Yes	579 (10.3)	867 (9.1)				
Prior to past-year DSM-5 drug-use use disorder			0.3127	1.7581	0.2844	0.0269
No	4710 (45.4)	3737 (39.4)				
Yes	338 (0.5)	699 (0.3)				
Prior to past-year DSM-5 mood disorder			0.2791	1.5592	0.3391	-0.0261
No	3784 (32.3)	2746 (47.3)				
Yes	1264 (21.0)	1690 (17.8)				
Prior to past-year DSM-5 anxiety disorder			0.9874	0.0003	0.2530	-0.0400
No	4170 (38.9)	3242 (34.2)				
Yes	878 (14.3)	1194 (12.6)				
Prior to past-year DSM-5 alcohol use disorder			0.2348	1.6022	0.3416	-0.0261
No	3716 (31.9)	2712 (28.6)				
Yes	1132 (21.4)	1724 (18.2)				

*Any DSM-5 personality disorder (antisocial, schizotypal, or borderline)

**Parents’ adverse experiences refer to a “yes” response to any of the following conditions: one of the parents going to jail, being hospitalized due to mental illness, attempting or committing suicide

***Any previous past-year mood disorder (depression, dysthymia, bipolar)

****Any previous past-year anxiety disorder (social anxiety, panic disorder with or without agoraphobia, specific phobia, generalized anxiety)

**Table 2:** Conditional effect of high (≥ 2) vs. low (0–1) stressful life events (SLE) on past-year DSM-5 Alcohol Use Disorder (AUD) criteria count at selected values of the polygenic risk score (PRS)

PRS	Stress Effect	SE	*p*
-1.8040	0.0429	0.1288	0.7390
-1.7129	0.0705	0.1182	0.5511
-1.6217	0.0980	0.1077	0.3629
-1.5306	0.1256	0.0974	0.1972
-1.4395	0.1531	0.0872	0.0792
-1.4050	0.1636	0.0834	0.0500
-1.3484	0.1807	0.0773	0.0195
-1.2573	0.2082	0.0678	0.0022
-1.1661	0.2358	0.0589	0.0001
-1.0750	0.2634	0.0509	0.0000

SE: standard error

PRS is z standardized (mean = 0, SD = 1); more positive values indicate higher polygenic liability for alcohol consumption (AUDIT C–derived). Each row gives the estimated difference in AUD criteria count between the high stress and low stress groups at the specified PRS value, with its standard error (SE) and two sided p value. The Johnson–Neyman significance threshold occurs at PRS ≈ −1.405 (p = 0.050): for PRS values greater than −1.405, the stress effect is statistically significant and increases with PRS

### Downstream analyses

For our NESARC-III primary analytical sample, we matched individuals in the exposure group who reported two or more past-year stressful events (*N* = 4436) with a control/low exposure group who reported one or zero past-year stressful events (*N* = 5048). To account for the complexity of the NESARC data survey, we performed the entire analysis in SAS using a set of orders that utilized Taylor series linearization. The two-sided level of significance was set at *p* < 0.05. We summarized the weighted rates of the clinical and demographic characteristics of the two groups.

First, we estimated the effects of SLEs on AUD severity using logistic regression analysis. The model controlled for the same matched variables for robust estimates and other variables known as risk factors that could not be matched, including age, marital status, and income. We estimated the effect of PRS or SLE on AUD severity using linear regression while controlling for the same variables.

For our second aim, we used the PROCESS macro within SAS to evaluate moderation probe interactions [46]. In this study, we evaluated whether PRS moderated the effect of SLEs on AUD severity. The interaction between stressful life events and the PRS was assessed based on its ability to predict AUD. A simple slope for the interaction between the PRS and high or low SLE was created. If there was a region of statistical significance within the continuous estimate of PRS that increases the probability of developing AUD, we used the Johnson–Neyman technique to determine the slope and highlight the significant interaction between PRS and stress. This technique allows for continuous measurements within the 95% confidence interval of the relationship between the change in PRS and the change in the probability of AUD.

## Results

### Sample characteristics

Our primary analytical sample included data from 11,815 NESARC-III participants who were self-reported as white (non-Hispanic), provided genetic samples, and were confirmed to have European genetic ancestry. The analysis further excluded participants who were not exposed to at least one alcoholic drink during their lifetime, yielding a total sample size of 10,775 participants. Following matching, data for 9,484 participants were included in the analysis, representing 5,048 individuals exposed to zero or one SLEs in the past year (low stress, control group) and 4,436 individuals exposed to two or more events in the past year (high stress, exposure group). Table 1 shows the clinical characteristics and diagnoses of the individuals in the low- and high-stress categories. Propensity score matching yielded data that were similar in age at first drinking, sex, birthplace, presence of parental adverse experiences, total childhood adversity experience scores, and prior to past-year mood, anxiety, and personality disorder diagnosis. Table 1 shows the standardized mean differences before and after matching for each variable.

### Effect of high-stress exposure on past-year AUD criteria count

Before controlling for potential confounders, a significant association was observed between high-stress and AUD criteria count (estimate: 0.62; 95%CI: 0.53–0.72; t-value: 13.12; *p* < 0.001). After controlling for potential confounders, the association remained statistically significant (estimate: 0.37; 95%CI: 0.27–0.48; t-value: 6.96; *p* < 0.001).

### Effect of PRS on past-year AUD criteria count

A significant association was observed between PRS and AUD criteria in matched data before adjustment for potential confounders (estimate: 0.48; 95%CI: 0.30–0.65; t-value: 5.38; *p* < 0.001) and after controlling for confounders (estimate: 0.23; 95%CI: 0.07–0.39; t value: 2.87; *p* = 0.01).

In a primary sensitivity analysis using a log-transformed AUD severity outcome, polygenic risk for AUD remained significantly associated with AUD severity (β = 0.074, SE = 0.026, *p* = 0.006). In a secondary analysis including the PRS × stress interaction, the interaction effect was attenuated under the transformed outcome, while the main effect of stress exposure remained significant.

### Interaction between PRS and stress

Firstly, there was a moderate positive survey-weighted correlation between polygenic risk for AUD and total past-year SLEs (*r* = 0.28), indicating that individuals with higher genetic liability tended to report greater exposure to recent stressors.

PRS moderated the relationship between the stress effect and the severity of AUD criteria, which was significant after controlling for all covariates (effect: 0.30; 95%CI: 0.06–0.54; t-value: 2.47; *p* = 0.01). Table 2 demonstrates that PRS at -1.35 significantly affected the development of AUD severity of criteria.

To evaluate whether the observed PRS × stress effect was sensitive to outcome scaling and to address potentially spurious G×E effects arising from unmodelled covariate-by-genetic and covariate-by-environment interactions, we conducted two additional analyses.

First, we re-estimated the primary survey-weighted regression model using a log-transformed AUD criteria outcome. In this model, the AUD PRS remained associated with higher AUD severity (b = 0.074, SE = 0.026, *p* = 0.006), indicating that the main genetic association was robust to outcome skewness and scaling.

Second, we re-estimated the PRS × stress model using the transformed outcome. Under survey-weighted regression, the PRS × stress interaction was attenuated and not statistically significant (b = − 0.002, SE = 0.040, *p* = 0.957). We then expanded this model by adding a full set of dummy-coded covariate-by-PRS and covariate-by-stress interaction terms (as recommended to reduce bias in G×E inference). In this expanded specification, the PRS × stress term remained non-significant (b = − 0.021, SE = 0.040, *p* = 0.594). A parallel PROCESS implementation using the same dummy-coded interaction set produced a consistent null PRS × stress estimate (b = 0.013, SE = 0.035, *p* = 0.714).

Fig. 1 illustrates the probability of higher AUD severity diverging depending on the interaction between stress category and PRS. The difference in the effect of PRS on the probability of increasing AUD severity expression was significant in the high-stress category

## Discussion

The current study demonstrated that polygenic liability of AUD depends on a well-known social risk factor, past-year exposure to stress. These findings provide the first statistical evidence from nationally representative data that stress exposure may potentiate the polygenic risk of AUD. Our study extends the literature on GE interactions in alcohol use, suggesting that stress exposure may exacerbate polygenic risk of AUD consumption traits. This GE statistical interaction model identified a positive interaction, indicating this population subgroup (E+) at a particularly high risk for AUD. Consequently, these individual populations (E + or GE+) may be a potential target group for interventions [47]

The significant effect of stress on AUD from our designed series of analyses is consistent with the previous research demonstrating the role of stress in the progression of substance use. We illustrated the role of stress experienced as a life event as a risk factor for the progression of AUD. Notably, stress exposure increases the risk of AUD severity, as SLE was seen to interact with AUD as a continuous variable of criteria count. This finding builds on two theoretical models that propose a role for stress in alcohol-related pathologies [48]. First, the tension reduction theory proposes that alcohol, a central nervous system depressant, is used to reduce tension following the experience of negative emotional states, such as fear, anxiety, distress, or depression [48, 49]. Social learning theory extends tension reduction notions by proposing that the stress-alcohol mechanism is moderated by coping behaviors, in which some individuals learn to drink in response to stress-inducing stimuli when they lack appropriate coping responses [48]. Diverse approaches have identified neurobiological links between stress and the risk of addiction [49]. Stress exposure results in a negative emotion-related state of alcohol craving [49]

Two additional robustness checks were conducted to evaluate the stability of the G×E findings. First, because AUD criteria counts are typically right-skewed, we repeated analyses using log-transformed AUD severity outcome. The association between the AUD PRS and AUD severity persisted under transformation, supporting robustness of the main genetic association to outcome scaling and skewness. Second, to address the possibility of spurious G×E effects due to unmodelled covariate-by-genetic and covariate-by-environment interactions, we re-estimated the PRS × stress model including a comprehensive set of covariate × PRS and covariate × stress terms. Under both the transformed outcome and the covariate-interaction-adjusted specification, the PRS × stress interaction was attenuated to near zero and was not statistically significant. Together, these results suggest that any PRS-dependent moderation by stress exposure is small and sensitive to model specification, and should be interpreted cautiously relative to the more stable main PRS association. The PRS was z-standardized, and covariates were placed on a common scale for propensity score estimation, reducing the likelihood that results reflect simple scaling artifacts

Overall, our preliminary findings are consistent with the extensive literature supporting the association between the PRS and AUD [14, 16–19]. These findings contribute to our understanding of the etiology of AUD by demonstrating the moderating role of polygenic risk in AUD severity. This is consistent with previous literature demonstrating the interactive effects of genetic and environmental factors on susceptibility to substance use [40]

Notably, the interactions between genes and psychosocial adversity influence alcohol consumption. For example, Laucht et al. (2009) found that alcohol-related gene expression changes in the LL genotype (5HTTLPR polymorphism) led to changes in alcohol intake and binge patterns, and the individuals in the high-stress groups drank more [49]. These findings show that multiple vulnerability factors can interact to produce specific behavioral patterns. However, statistical GE studies of AUD focusing on stress as a moderator have mostly relied on twin studies and specific genes rather than a polygenic approach [14]

We report a modest positive, survey-weighted correlation between PRSs and SLEs. This association is consistent with prior twin research demonstrating gene–environment correlation (rGE), whereby genetic liability to psychopathology contributes not only to disorder risk but also to exposure to stressful life events, particularly those that are behaviorally or interpersonally mediated [50]. Twin studies have shown that many “dependent” stressors are partly heritable and may arise through evocative processes (genetically influenced traits eliciting adverse responses from the environment) or active processes (selection into higher-risk contexts), rather than representing purely random environmental shocks (50–52]. Accordingly, the observed correlation between PRS and SLEs suggests that stress exposure in this sample may partially reflect genetically influenced selection or evocation mechanisms, which should be considered when interpreting PRS × SLE interaction effects as statistical moderation rather than strictly causal environmental effects.

### Limitations and future directions

Our study had some limitations. First, given the cross-sectional and retrospective nature of the NESARC-III data, we could not confirm whether SLEs preceded the onset of AUD criteria to confirm the causal pathway. Controlling for the prior-to-past year AUD clinical variable was a measure taken to better isolate the relationship between SLE and current AUD symptoms. Also, we were limited by further clinical data present within the NESARC-III dataset. The SLE scale reported in the NESARC-III does not include contextual information regarding responses to stressors, and the SLE items are weighted similarly. The stress response threshold may vary among individuals. Furthermore, a within-family comparison that matches individuals for familial confounds, including some background genetic risk, would further strengthen the credibility of propensity score matching.

Secondly, our results are preliminary for investigating the clinical utility of PRS and may not be generalized to non-European ancestry. Our analysis was limited to self-reported White (non-Hispanic) participants of EA and included only genetic samples that were in concordance between self-reported race and genetic ancestry. This is consistent with the samples used in the base GWAS data. Therefore, consistent replication across diverse populations is required in future studies. Regarding GWAS selection, we agree that variants contributing to GxE effects may differ from those driving main genetic effects on AUD, and we highlight this as an important direction for future research. Specifically, GWEIS sample sizes are often substantially smaller than main-effect GWAS, and interaction effects may be ancestry specific. A key contribution of the present study is to demonstrate both the promise and current limitations of integrating polygenic risk with environmental stressors using existing GWAS resources. Our findings underscore the need for larger, ancestrally diverse GWEIS efforts that can support the development of interaction-informed PRS and ultimately advance risk modeling toward clinical utility. 

To our knowledge, this is the first study to demonstrate the moderating effect of polygenic liability (as assessed using the PRS) on the effect of SLE exposure on the progression of DSM-5 AUD. This GE interaction model provides evidence that polygenic risk can potentiate the risk of high-stress exposure for the development of AUD in a nationally representative sample. Taken together, these findings underscore the importance of integrating genetic and environmental information to improve risk stratification, guide targeted prevention efforts, and inform early intervention strategies for individuals most vulnerable to stress-related escalation of alcohol misuse.

## Data Availability

The datasets analyzed during the current study are available in the NESARC/NIH repository, [dbGaP Study](https:/www.ncbi.nlm.nih.gov/projects/gap/cgi-bin/study.cgi? study_id=phs001590.v2.p1).
